# Prognostic significance of C-reactive protein in patients with intermediate-risk metastatic renal cell carcinoma treated with molecular targeted therapy

**DOI:** 10.3892/ol.2014.2207

**Published:** 2014-06-02

**Authors:** JUN TEISHIMA, KOHEI KOBATAKE, TETSUTARO HAYASHI, YASUYUKI SENO, KENICHIRO IKEDA, HIROTAKA NAGAMATSU, KEISUKE HIEDA, KOICHI SHOJI, KATSUTOSHI MIYAMOTO, SHOGO INOUE, KANAO KOBAYASHI, SHINYA OHARA, MITSURU KAJIWARA, AKIO MATSUBARA

**Affiliations:** Department of Urology, Institute of Biomedical and Health Sciences, Integrated Health Sciences, Hiroshima University, Hiroshima 734-8551, Japan

**Keywords:** C-reactive protein, renal cell carcinoma, prognostic factor

## Abstract

The present study aimed to investigate the impact of pre-treatment C-reactive protein (CRP) levels on the prediction of prognosis in patients with metastatic renal cell carcinoma (mRCC), who were classified as intermediate-risk patients using the Memorial Sloan Kettering Cancer Center (MSKCC) risk classification and who received molecular targeted therapy. The oncological outcome of 140 patients with mRCC who underwent molecular targeted therapy was analyzed. Patients were divided into favorable-, intermediate- and poor-risk groups (groups F, I and P, respectively) based on the MSKCC risk classification. The patients in group I were then further classified into two groups based on pre-treatment serum CRP levels. The overall survival (OS) rates of the patients in these groups were then assessed. The OS rate of the patients in group I with normal pre-treatment CRP levels was found to be significantly increased compared with that of patients with high pre-treatment CRP levels (P<0.0001), while there was no significant difference in the OS rate in the patients with normal pre-treatment CRP levels in group I compared with those in group F. Multivariate analyses revealed that high pre-treatment CRP levels were an independent prognostic factor for OS in the patients in group I (P<0.0001; hazard ratio, 3.898). Thus, pre-treatment CRP levels may be a candidate predictor for OS in patients with intermediate-risk mRCC.

## Introduction

In the European population, renal cell carcinoma (RCC) accounts for 2% of all new cancer cases and 25% of patients with RCC have metastases at initial presentation (1). The majority of cases of metastatic RCC (mRCC) are refractory for chemotherapy, and immunotherapy using interferon and/or interleukin (IL) has been effective for only a small proportion of patients with mRCC. Recently, molecular targeted therapies have been widely used and the therapeutic strategy for mRCC has changed markedly as randomized control trials have demonstrated the efficacy and safety of these drugs for the treatment of mRCC (2–4). Three tyrosine kinase inhibitors, sorafenib, sunitinib and axitinib, as well as two mammalian target of rapamycin inhibitors, everolimus and temsirolimus, have been approved for the treatment of patients with mRCC in Japan, and the clinical outcomes of these agents have been reported (5–9).

One of the most well-established classification systems for patients with mRCC is the Memorial Sloan Kettering Cancer Center (MSKCC) system reported by Motzer *et al* (10) in 1999 and modified in 2002 (11). In the modified classification system, patients are classified into three groups based on the following five risk factors for short survival: Low Karnofsky performance status (<80%), low hemoglobin levels (<the lower limit of normal), high serum lactate dehydrogenase levels (>1.5-fold the upper limit of normal), high corrected calcium levels (>10 mg/dl) and short time from diagnosis to the initiation of targeted therapy (<1 year). Internal and external validation (11,12) in numerous clinical studies in Europe and America has found this risk classification system to be useful for analyzing the prognosis of patients with mRCC. Although the MSKCC risk classification system is useful, between 53 and 70% of all patients with mRCC have been classified into the intermediate-risk group (11–13). Thus, patients with better prognoses may be included in same group as those with worse prognoses. To predict the prognosis of each patient, an additional factor which classifies the intermediate risk group into two subgroups is required.

Previous studies have demonstrated that there are several prognostic factors for advanced RCC other than the five used in the MSKCC risk classification system, including serum C-reactive protein (CRP) levels (14–18), metastasis status (12,19,20) and previous treatments (21–23). Thus, the present study aimed to investigate the impact of these factors and other factors on the prognosis of patients with intermediate-risk mRCC receiving molecular targeted therapy.

## Patients and methods

### Patients

A total of 146 patients underwent molecular targeted therapy at the Institute of Biomedical and Health Sciences (Hiroshima University, Hiroshima, Japan) and other hospitals in the Hiroshima prefecture between 2007 and 2011. Six of the patients were excluded from the present study, as their pre-treatment serum CRP levels were not known. The remaining 140 patients were retrospectively classified into favorable-, intermediate- and poor-risk groups (groups F, I and P, respectively). Group I was further classified according to age, metastasis status, prior nephrectomy, choice of first-line drug treatment and pre-treatment serum CRP levels. The overall survival (OS) rate of the patients in each subgroup was then compared with that in groups F and P. The study was approved by the ethics committee of Hiroshima University (Hiroshima, Japan).

### Statistical analysis

In each group, the OS rate from the initiation of molecular targeted therapy to the date of mortality was determined using the Kaplan-Meier method, and differences between groups were analyzed using the log-rank test. χ^2^ analysis was used for categorical variables. For multivariate analyses, the Cox proportional-hazards regression model was used. All statistical analyses were performed using the StatView 5.0 software package (SAS Institute, Inc., Cary, NC, USA) and P<0.05 was considered to indicate a statistically significant difference.

## Results

The cohort included in the present study consisted of 140 consecutive patients who underwent molecular targeted therapy for mRCC. The characteristics of the patients are shown in Table I. In total, 118 (84.3%) patients were male. Twenty-two patients (15.7%) were classified into group F, 95 (67.9%) were classified into group I and 23 (16.4%) were classified into the group P. In group I, the percentage of patients with two or more metastatic organs was significantly reduced (P=0.0001) compared with that in group P, and the percentage of patients with pre-treatment CRP levels >0.3 mg/dl was also significantly reduced (P=0.0025) compared with that in group P. Furthermore, in group I, the percentage of patients who had previously undergone nephrectomy was significantly increased (P<0.0001) compared with that in group P.

**Figure 1:**
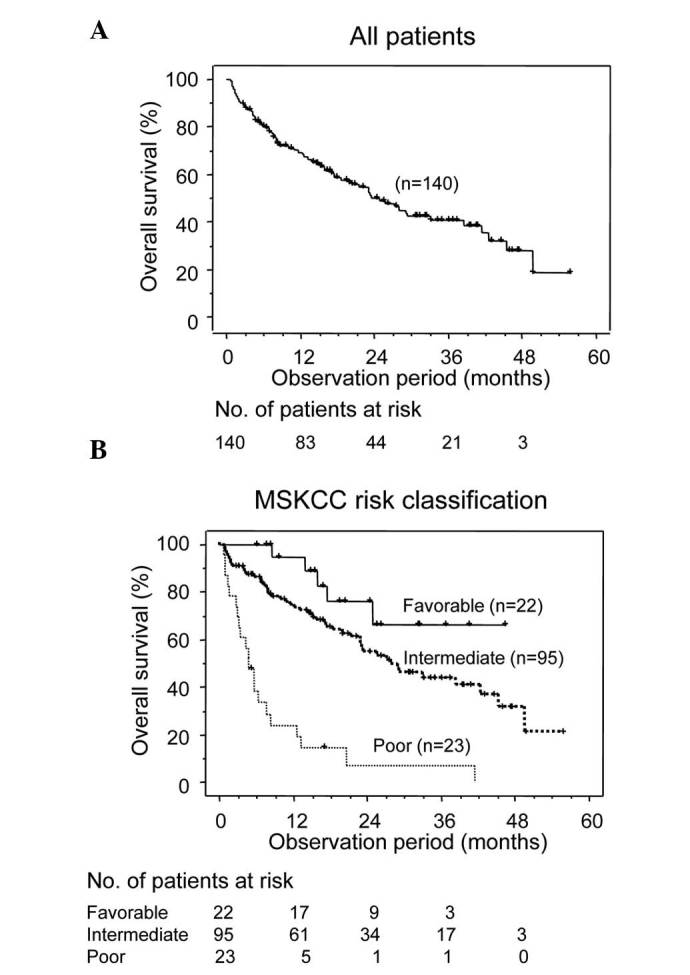
Overall survival curves for (A) all 140 patients with metastatic renal cell carcinoma and (B) the same patients stratified using the MSKCC risk classification. MSKCC, Memorial Sloan Kettering Cancer Center.

**Figure 2:**
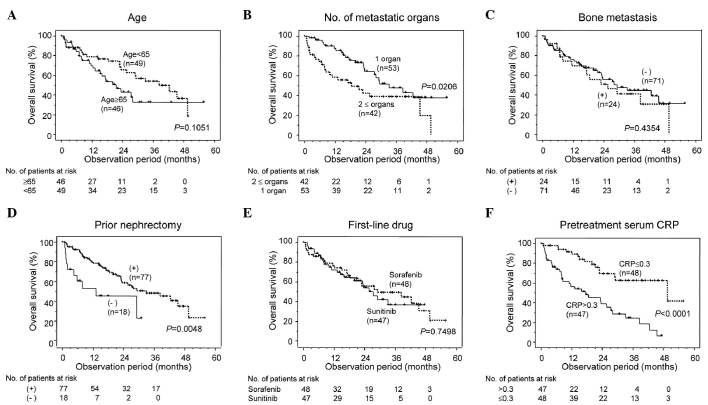
Overall survival rates of 95 patients with metastatic renal cell carcinoma in the intermediate-risk group who were further classified according to (A) age, (B) the number of metastatic organs, (C) bone metastasis, (D) prior nephrectomy, (E) the choice of first-line drug treatment and (F) pre-treatment serum CRP levels. CRP, C-reactive protein.

The OS curve for the entire cohort of patients is shown in Fig. 1A. During the follow-up period, which was a median of 15.6 months, 70/140 (50.0%) patients succumbed due to RCC and 3/140 (2.1%) patients succumbed due to other causes. The 1-, 2- and 3-year OS rates for the entire cohort were 69.3, 49.9 and 40.6%, respectively. The OS curves for the three MSKCC risk groups are shown in Fig. 1B. The 1-, 2- and 3-year OS rates for group F (94.7, 75.9 and 66.4%, respectively) and group I (74.3, 54.4 and 43.7%, respectively) were significantly increased (P<0.0001) compared with those for group P (19.1, 7.2 and 0%, respectively). To elucidate the prognosis of the patients in group I more accurately, the patients in group I were further classified into two subgroups based on age, serum CRP levels prior to commencing molecular targeted therapy, prior nephrectomy, first-line drug treatment, bone metastasis and the number of metastatic organs. When CRP levels ≤0.3 mg/dl were considered to be normal, the 1-, 2- and 3-year OS rates of the patients in the normal-CRP subgroup (91.3, 69.3 and 62.3%, respectively) were significantly increased (P<0.0001) compared with those of the patients in the high-CRP subgroup (56.5, 38.7 and 24.6%, respectively) (Fig. 2F).

When patients in group I were classified into two subgroups based on the five remaining factors, the OS of the patients with two or more metastatic organs was found to be significantly decreased (P=0.0206) compared with that of the patients with one or no metastatic organs, and the OS of the patients with no prior nephrectomy was significantly decreased (P=0.0048) compared with that of those who had previously undergone nephrectomy (Fig. 2B and D). Multivariate analyses revealed that pre-treatment serum CRP levels and prior nephrectomy were independent prognostic factors for OS in the patients in group I (P<0.0001 and P=0.0313, respectively) (Table II).

The OS of the patients in the high-CRP subgroup of group I was significantly decreased compared with that of the patients in group F (P=0.0126), while it was significantly increased compared with that of the patients in group P (P=0.0009). Furthermore, OS rates were not observed to differ significantly between the patients in group F and the patients in the normal-CRP subgroup of group I (P=0.7556) (Figs. 1B and 2F). These data show that the patients in group I could be divided into two subgroups with different prognoses based on pre-treatment CRP levels.

## Discussion

The present study elucidated the efficacy of using pre-treatment CRP levels to further classify the MSKCC risk classification’s intermediate-risk group into two subgroups with significantly different prognoses in the molecular targeted therapy era. Molecular targeted therapy has markedly changed the treatment strategy for mRCC; therefore, establishing an enhanced risk classification for patients with mRCC is important. The MSKCC risk classification system is widely used globally. Patil *et al* (19) analyzed prognostic factors in a randomized study using sunitinib or interferon-α and demonstrated the use of the same five factors used in the MSKCC risk classification system. This study suggests that the MSKCC risk classification system is useful in the molecular targeted therapy era, even though it was based on data of patients with mRCC treated during the cytokine therapy era. However, for Japanese patients with mRCC, there are certain differences with regard to the distribution of each group in the MSKCC risk classification, and an increased OS time was reported for patients with mRCC in each group (13). The intermediate-risk group (group I) is the largest of the three risk groups in numerous studies, including the present study (Table I) (10–13). In the present study, while there were a number of significant differences in background and OS rate between the patients in group I compared with those in group P, there were not as many differences between the patients in group I and group F (Table I, Fig. 1). Thus, group I may include patients with quite different prognoses and should be divided into two subgroups in order to determine the prognosis of the patients more accurately.

Previous studies have demonstrated several prognostic factors for advanced RCC besides those used in the MSKCC risk classification system. Bone metastasis has been reported to be a predictive factor associated with poor prognosis (20) and Mekhail *et al* (12) demonstrated the importance of the number and site of metastases in the study for external validation of the MSKCC risk classification system. Furthermore, several studies have reported the impact of cytoreductive nephrectomy as an independent prognostic factor for OS in patients with mRCC (21–23). Serum CRP levels have been suggested to be one of the most important prognostic factors for advanced RCC (14). Pre-operative serum CRP levels have been reported to be a predictor for metastasis and mortality following radical nephrectomy in patients with localized RCC (15,16). In addition, in patients with mRCC, CRP kinetics have been demonstrated to be a predictor for clinical course (17).

In the present study, the patients in group I were classified into two subgroups based on age at initial presentation, serum CRP levels prior to commencing molecular targeting therapy, previous nephrectomy, the existence of bone metastases and the number of metastatic organs. The OS rate of the patients whose pre-treatment CRP levels were ≤0.3 mg/dl was found to be significantly increased compared with that of the patients whose pre-treatment CRP levels were >0.3 mg/dl. In addition, the OS rate of the patients who had previously undergone nephrectomy was significantly increased compared with that of the patients who had not undergone nephrectomy (Fig. 2). Furthermore, high pre-treatment serum CRP levels and prior nephrectomy were identified to be independent prognostic factors for OS rate in patients with mRCC (Table II). CRP is one of the most representative acute-phase reactants produced primarily in the liver in response to inflammatory reactions through interleukin (IL)-6 signaling (24). Elevated CRP levels are observed during infection, cardiovascular diseases, diabetes and malignancies (24). Several studies have reported an association between elevated serum CRP levels and malignant diseases other than RCC, including colorectal cancer (25), lung cancer (26) and urothelial cancer (27). The mechanism through which a systemic inflammatory response, indicated by elevated CRP levels, reduces OS rates in patients with mRCC is still unclear. However, certain relevant experimental data have been reported (28,29). A previous study showed that the release of cytokines and growth factors in an inflammatory response stimulates tumor growth (28). Furthermore, it has been demonstrated that renal cancer cells may produce IL-6, which may promote the growth of renal cancer cells in an autocrine manner (29). These reports suggest that CRP may have an important role in the progression of RCC. Measuring serum CRP levels is useful, simple, inexpensive and reproducible. Thus, serum CRP levels may be a powerful biomarker for mRCC.

One limitation of the present study is that it is retrospective; therefore, patient selection bias may exist. For example, the patients enrolled in the present study included those in whom mRCC was first diagnosed and those in whom mRCC recurred following radical nephrectomy. In the present study, ‘prior nephrectomy’ refers to both cytoreductive nephrectomy for mRCC and radical nephrectomy prior to recurrence. Further prospective investigations are required to confirm the potential of pre-treatment serum CRP levels to be used to predict prognosis in patients with intermediate-risk mRCC. In conclusion, the present study demonstrated the role of serum CRP levels as a prognostic factor in patients with mRCC in the intermediate-risk group, prior to being treated with molecular targeted therapy. Further classifying the patients with different prognoses in the intermediate-risk group may help determine the prognosis of patients with mRCC more accurately. Further studies are required to establish an enhanced strategy for the use of novel molecular targeted agents, based on more accurate risk classification, involving the consideration of patient CRP levels.

## Figures and Tables

**Table I:** Characteristics of 140 patients with metastatic renal cell carcinoma who underwent molecular targeted therapy.

	MSKCC risk			
		
Parameter	Favorable	Intermediate	Poor	Total
No. patients	22	95	23	140
Age, range (median)	46–81 (63)	40–85 (64)	39–78 (62)	39–85 (64)
Gender, n (%)				
Male	19 (86.4)	82 (86.3)	17 (73.9)	118 (84.3)
Female	3 (13.6)	13 (13.7)	6 (26.1)	22 (15.7)
Histological type, n (%)				
Clear	19 (86.4)	67 (70.5)	7 (30.4)	93 (66.4)
Non-clear	2 (9.1)	6 (6.3)	3 (13.0)	11 (7.9)
Unknown	1 (4.5)	22 (23.2)	13 (56.5)	36 (25.7)
No. metastatic organs, n (%)				
1	11 (50.0)	53 (55.8)^a^	2 (8.7)	66 (47.1)
≥2	11 (50.0)	42 (44.2)	21 (91.3)	74 (52.9)
Bone metastasis, n (%)				
Yes	5 (22.7)	24 (25.3)	5 (21.7)	34 (24.3)
No	17 (77.3)	71 (74.7)	18 (78.3)	106 (75.7)
Prior nephrectomy, n (%)				
Yes	22 (100.0)	77 (81.0)^a^	7 (30.4)	106 (75.7)
No	0 (0.0)	18 (19.0)	16 (69.6)	34 (24.3)
Pre-treatment CRP levels, n (%)				
≤0.3 mg/dl	16 (72.7)	48 (50.5)	3 (13.0)	67 (47.9)
>0.3 mg/dl	6 (27.3)	47 (49.5)^a^	20 (87.0)	73 (52.1)

a P<0.05 vs. poor-risk group.

MSKCC, Memorial Sloan Kettering Cancer Center; CRP, C-reactive protein.

**Table II:** Multivariate analyses of the association between various paramaters and overall survival in patients with intermediate-risk metastatic renal cell carcinoma.

	Univariate analysis	Multivariate analysis		
		
Parameter	P-value (log-rank)	HR	95% CI	P-value
Number of metastatic organs (1 vs. ≥2)	0.0206	0.561	0.302–1.043	0.0677
Bone metastasis	0.4354	-	-	-
Prior nephrectomy	0.0048	2.394	1.082–5.298	0.0313
First-line drug	0.7498	-	-	-
Pre-treatment CRP level (normal vs. abnormal)	<0.0001	3.898	2.062–7.370	<0.0001
Age at diagnosis (years; <65 vs. ≥65)	0.1051	-	-	-

CRP, C-reactive protein; HR, hazard ration; CI, confidence interval.
